# Hippocampal-Brainstem Connectivity Associated with Vagal Modulation after an Intense Exercise Intervention in Healthy Men

**DOI:** 10.3389/fnins.2016.00145

**Published:** 2016-04-07

**Authors:** Karl-Jürgen Bär, Marco Herbsleb, Andy Schumann, Feliberto de la Cruz, Holger W. Gabriel, Gerd Wagner

**Affiliations:** ^1^Psychiatric Brain and Body Research Group, Department of Psychiatry and Psychotherapy, University Hospital JenaJena, Germany; ^2^Clinical Exercise Physiology, Department of Sports Medicine and Health Promotion, Friedrich-Schiller-University of JenaJena, Germany

**Keywords:** exercise, vagal, cognition, hippocampus, heart, brainstem, physical fitness, central autonomic network

## Abstract

Regular physical exercise leads to increased vagal modulation of the cardiovascular system. A combination of peripheral and central processes has been proposed to underlie this adaptation. However, specific changes in the central autonomic network have not been described in human in more detail. We hypothesized that the anterior hippocampus known to be influenced by regular physical activity might be involved in the development of increased vagal modulation after a 6 weeks high intensity intervention in young healthy men (exercise group: *n* = 17, control group: *n* = 17). In addition to the determination of physical capacity before and after the intervention, we used resting state functional magnetic resonance imaging and simultaneous heart rate variability assessment. We detected a significant increase of the power output at the anaerobic threshold of 11.4% (*p* < 0.001), the maximum power output Pmax of 11.2% (*p* < 0.001), and VO2max adjusted for body weight of 4.7% (*p* < 0.001) in the exercise group (EG). Comparing baseline (T0) and post-exercise (T1) values of parasympathetic modulation of the exercise group, we observed a trend for a decrease in heart rate (*p* < 0.06) and a significant increase of vagal modulation as indicated by RMSSD (*p* < 0.026) during resting state. In the whole brain analysis, we found that the connectivity pattern of the right anterior hippocampus (aHC) was specifically altered to the ventromedial anterior cortex, the dorsal striatum and to the dorsal vagal complex (DVC) in the brainstem. Moreover, we observed a highly significant negative correlation between increased RMSSD after exercise and decreased functional connectivity from the right aHC to DVC (*r* = −0.69, *p* = 0.003). This indicates that increased vagal modulation was associated with functional connectivity between aHC and the DVC. In conclusion, our findings suggest that exercise associated changes in anterior hippocampal function might be involved in increased vagal modulation.

## Introduction

Regular physical exercise leads to a wide range of physiological and psychological changes. Cardiovascular adjustments due to a shift in central autonomic control and remodeling of the heart are the most prominent features of exercise training (Negrao et al., 1993; Brum et al., 2000). It has been suggested that a reduced sympathetic modulation and an arising parasympathetic dominance might be caused by adaptations of peripheral and central regulatory systems (Sugawara et al., 2001). However, the underlying neuroplastic changes of autonomic control in the brain during adjustments to regular exercise are not well understood. Neural circuitries involved in autonomic responses to exercise are likely to incorporate areas in the brainstem and supramedullary centers. Cardiovascular centers in the brainstem work through various cardiovascular reflex mechanisms such as baroreflex, chemoreflex, and cardiopulmonary reflex (Dampney, 1994). Afferent fibers of these cardiovascular reflexes terminate in nuclei of the solitary tract. The efferent sympathetic reflex component is determined by neurons in the caudal and rostral ventrolateral medulla. These neurons contribute to the maintenance of blood pressure and heart rate (HR) by signaling to the intermediolateral column of the spinal cord. Two further medullary areas contain preganglionic parasympathetic neurons: the nucleus ambiguous and the dorsal motor nucleus of the vagus nerve. Both mediate the efferent parasympathetic component of above described reflexes (McAllen, 1976; McAllen and Spyer, 1976; Taylor et al., 2001). These preganglionic parasympathetic neurons might either show cardiac or respiration-related activity. Brainstem nuclei and pathways receive modulatory inputs from supramedullary centers such as the insula, thalamus, hypothalamus, amygdala, parietal and cingulate regions, or the medial prefrontal region (Owens and Verberne, 2000). Various studies have shown the involvement of these brain areas in the autonomic regulation at rest and during cognitive or emotional strains by means of functional brain imaging (Critchley, 2005; Lane et al., 2009; Ziegler et al., 2009; Shoemaker et al., 2015). It has been assumed that processing various autonomic functions is generated by a network interaction showing specificity for task and autonomic division (Beissner et al., 2013). An additional region, which is of particular interest for exercise interventions and changes of autonomic function, is the hippocampus (HC). The hippocampus is a medial temporal lobe structure, which is amongst others involved in episodic memory and spatial navigation. Moreover, animal and human studies suggest an anterior-posterior subdivision of the hippocampus into cognitive or affective functional domains (Fanselow and Dong, 2010; Poppenk et al., 2013). In rodents, the dorsal part of the hippocampus, which corresponds to the posterior hippocampus (pHC) in primates, is considered to be mainly involved in memory and learning processes through tight projections to the anterior cingulate and retrosplenial cortex (posterior cingulate cortex in primates) (Moser et al., 1995; Fanselow and Dong, 2010). In humans, single-unit recordings and neuroimaging studies indicated greater pHC involvement in the recollection of detailed spatial (Nadel et al., 2013) and verbal information (Ludowig et al., 2008). The ventral part of the hippocampus, which corresponds to the anterior hippocampus (aHC) in primates, is anatomically strongly connected with the amygdala, nucleus accumbens (NAc), insula and the medial prefrontal cortex (Tannenholz et al., 2014). Furthermore, it forms, together with projections to the hypothalamic nuclei, a circuitry involved in emotional experiences and control of affective states (Cenquizca and Swanson, 2007; Fanselow and Dong, 2010). In human subjects, neuroimaging studies revealed that the fMRI activation of the aHC was associated more strongly with reward-directed behavior (Viard et al., 2011) and with memory for emotional material (Dolcos et al., 2004; Murty et al., 2010).

The latter hippocampal region is particularly sensitive to changes induced by physical activity (van Praag et al., 1999; Pereira et al., 2007; Erickson et al., 2011; Strange et al., 2014). Apart from animal studies showing that neurogenesis in the HC is associated with physical exercise (van Praag et al., 1999), Erickson et al. (2011) provided evidence for an association between increased volume in the right aHC and performance on a memory task following an exercise intervention in humans. While Erickson et al. (2011) described results in older sedentary subjects, our group has found comparable findings in the right aHC after a high intensity exercise intervention in young students (Wagner et al., 2015). We found very similar associations between aHC volume, fitness and the brain derived neurotrophic factor in our subjects although an overall decrease in hippocampal volume was observed. We have therefore suggested that individual response characteristics to exercise, such as age, gender, genetic background, or the inflammatory response, might be important for exercise induced changes in brain structure and function (Wagner et al., 2015). In the present study we aim to elucidate whether functional and structural changes in the right aHC might be related to an increased vagal modulation following the exercise intervention. Previous studies have shown tight neural connections between the aHC and brainstem autonomic nuclei (Westerhaus and Loewy, 2001; Castle et al., 2005). Radna and Radna and MaClean (1981) have found that vagal nerve stimulation influences hippocampal neurons after 100–200 ms delay. In addition, vagal nerve stimulation enhances memory, which is a well-documented hippocampal function both in rats and humans (Clark et al., 1995, 1999). Furthermore, it has been assumed that A2 noradrenergic and non-catecholaminergic neurons of the medial solitary tract in the medulla are linked via a multisynaptic pathway to the ventral CA1 hippocampal field (Castle et al., 2005). Apart from that, regions in the aHC are known to influence the medial prefrontal cortex, most major subfields of the amygdala and various subnuclei of the hypothalamus, including the anterior hypothalamus and lateral hypothalamus (Tannenholz et al., 2014; Blessing et al., 2016). These connections are thought to provide the anatomical basis for the close involvement of aHC in the regulation of emotion and stress. Interestingly, previous studies showed an inverse relationship between hippocampal activation and cardiovascular arousal. An electrical stimulation of the aHC elicited a depression of cardiovascular activation (Ruit and Neafsey, 1990). Most intriguingly, cardiovascular responses in this electrical stimulation model required an intact medial prefrontal cortex.

Thus, the present study used resting state functional magnetic resonance imaging (rs-fMRI) in a controlled design in order to investigate whether an increased vagal modulation after a 6-week intense exercise intervention is related to changes in functional connectivity patterns of the anterior hippocampus. Based on our recently published results of structural changes in the right aHC after an exercise intervention (Wagner et al., 2015) and on known anatomical connectivity patterns, we hypothesized significant changes in the functional connectivity of the right aHC to cortical/subcortical brain regions, in particular to the medial prefrontal cortex, amygdala as well as to cardiovascular brainstem regions.

## Methods

### Subjects

**Figure 1:**
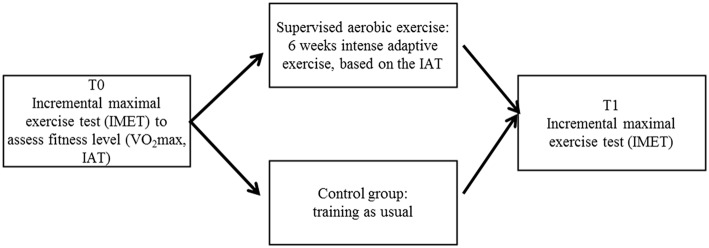
**Flow chart of the study**.

Thirty-four male students were recruited from the local university to participate in the present study for a total period of 8 weeks. We allocated 17 subjects to the physical training condition. To control for effects regarding the exercise intervention, 17 subjects who were matched for age, gender, body mass index (BMI) and maximum oxygen uptake (VO_2max_) were assigned to the control condition (no additional specific training) (Figure 1, Table 1). The exercise group was assigned to a 6-week controlled training intervention including 1 week beforehand and one afterwards for pre- and post-training investigations. Participants had no present or past history of any clinically significant disorders as assessed by their stated medical history, a physical examination and by applying the short form of the structured diagnostic interview for DSM-IV psychiatric disorders M.I.N.I. International Neuropsychiatric Interview (Sheehan et al., 1998). According to the modified version of the Annett's handedness inventory (Briggs and Nebes, 1975) the participants were right-handed and reported normal or corrected to normal vision. Informed written consent was obtained in accordance with the protocols approved by the ethics committee of the Jena University Hospital in accordance with the ethical guidelines of the Helsinki Declaration of 1975 (as revised in 1983) before conducting the study. Furthermore, each subject received 50 Euro for participating in the study.

**Table 1:** **Epidemiological data of participants**.

**Parameters**	**Control group**	**Exercise group**	***p***
	**Mean ± SD**	**Mean ± SD**	
Participants	*n* = 17	*n* = 17	n.a.
Age (years)	23.7 | 1.7	25.0 | 3.3	n.s.
Body-Mass-Index_*T*0_(kg/m^2^)	23.8 | 2.1	23.8 | 2.0	n.s.
P_maxT0_ (W)	318 | 41	295 | 23	n.s.
P_maxT1_ (W)	316 | 43	329 | 20	n.s.
P_IAnTT0_ (W)	206 | 26	185 | 25	<0.05
P_IAnTT1_ (W)	214 | 26	204 | 18	n.s.
VO_2maxT0_ (ml/kg/min)	44.6 | 6.3	45.9 | 4.8	n.s.
VO_2maxT1_ (ml/kg/min)	44.7 | 5.8	48.3 | 4.5	n.s.
Lactate_maxT0_ (mmol/l)	11.2 | 2.9	12.2 | 2.0	n.s.
Lactate_maxT1_ (mmol/l)	10.4 | 2.7	13.2 | 1.6	<0.001

*T0, data obtained before training intervention; T1, data obtained after training intervention; P, power output; P_IAnT_, power output at the individual anaerobic threshold; VO_2_, oxygen uptake*.

### Physical training

The training group had to complete three exercise sessions per week over a period of 6 weeks. Training was performed on a bicycle ergometer (Ergometrics 900, Ergoline, Bitz, Germany). A standardized warm-up period (5 min) was followed by 50 min of training and a cool-down period of another 5 min. Before the exercise intervention, an incremental maximal exercise test (IMET) was performed to determine the individual aerobic and anaerobic threshold (IAnT) as well as further fitness parameters to account for individual differences in physical fitness and to allow training at comparable intensities. In the beginning, the intensity of the training session matched the power output of 85% regarding the interval between the individual aerobic threshold and the IAnT. This was equivalent to 77 ± 9% of pretraining VO_2max_ (ranging from 60 to 88%). After 3 weeks of training, the intensity was again adjusted to the participants' individual physical capacity. One week after the accomplishment of the physical training program, fitness parameters were re-assessed during IMET to analyze the effect of the program.

### Incremental maximal exercise test (IMET)

A bicycle ergometer (SRM System, Schoberer Radmesstechnik, Jülich, Germany) was used to perform the IMET. The incremental bicycle protocol started after a resting period of 5 min at 50 W and increased by 50 W every 3 min until volitional exhaustion. Maximum lactate levels, maximal heart rate and the respiratory exchange ratio were assessed. Moreover, breath-by-breath gas exchange measurements were carried out throughout the test (Ganshorn, Medizin Electronic GmbH, Niederlauer, Germany) and transferred to Microsoft Excel for further analysis. VO_2_ data were time-averaged using 10 s intervals to assess VO_2max_ that corresponded to the highest VO_2_ over three intervals. P_max_ was defined as the maximal achieved power output sustained for 3 min. P_max_ was linearly interpolated when participants were not able to cycle to the end of the final 3-min interval.

Capillary blood samples for lactate measurements (Enzymatic-Amperometric Measuring System, Eppendorf, Hamburg, Germany) were taken at the end of each stage as well as 1, 3, 5, 7, and 10 min after accomplishing the exercise. Individual aerobic thresholds and IAnTs were determined using the lactate-power output plot. Special software (ERGONIZER, Freiburg, Germany) was used for the investigator-independent calculation of individual aerobic threshold, which represents the first increase in blood lactate concentrations above resting state values during incremental exercise. The IAnT describes the maximal lactate steady state and refers to the exercise intensity above which a continuous increase in blood lactate is unavoidable. The IAnT was calculated according to the method described by Stegmann et al. (1981). The Borg 6-to-20 scale was used to assess the degree of subjective effort perceived by the participants.

### Physiological parameters assessed during IMET

During the cycling exercise, the heart rate was continuously recorded throughout the testing session using a telemetric HR monitor (RS800CX, Polar Electro, Kempele, Finland). Time series exported from the device were post-processed by adaptive filtering (Wessel et al., 2000). The physiological recordings were assessed under similar conditions, i.e., sitting on a bicycle ergometer before (T0) and after physical exercise intervention (T1).

To analyze temporal evolution of parasympathetic cardiac activity, the RMSSD (root mean square of successive heart beat interval differences) was calculated in a sliding window of 256 heart beat intervals with 192 intervals overlap.

**Figure 2:**
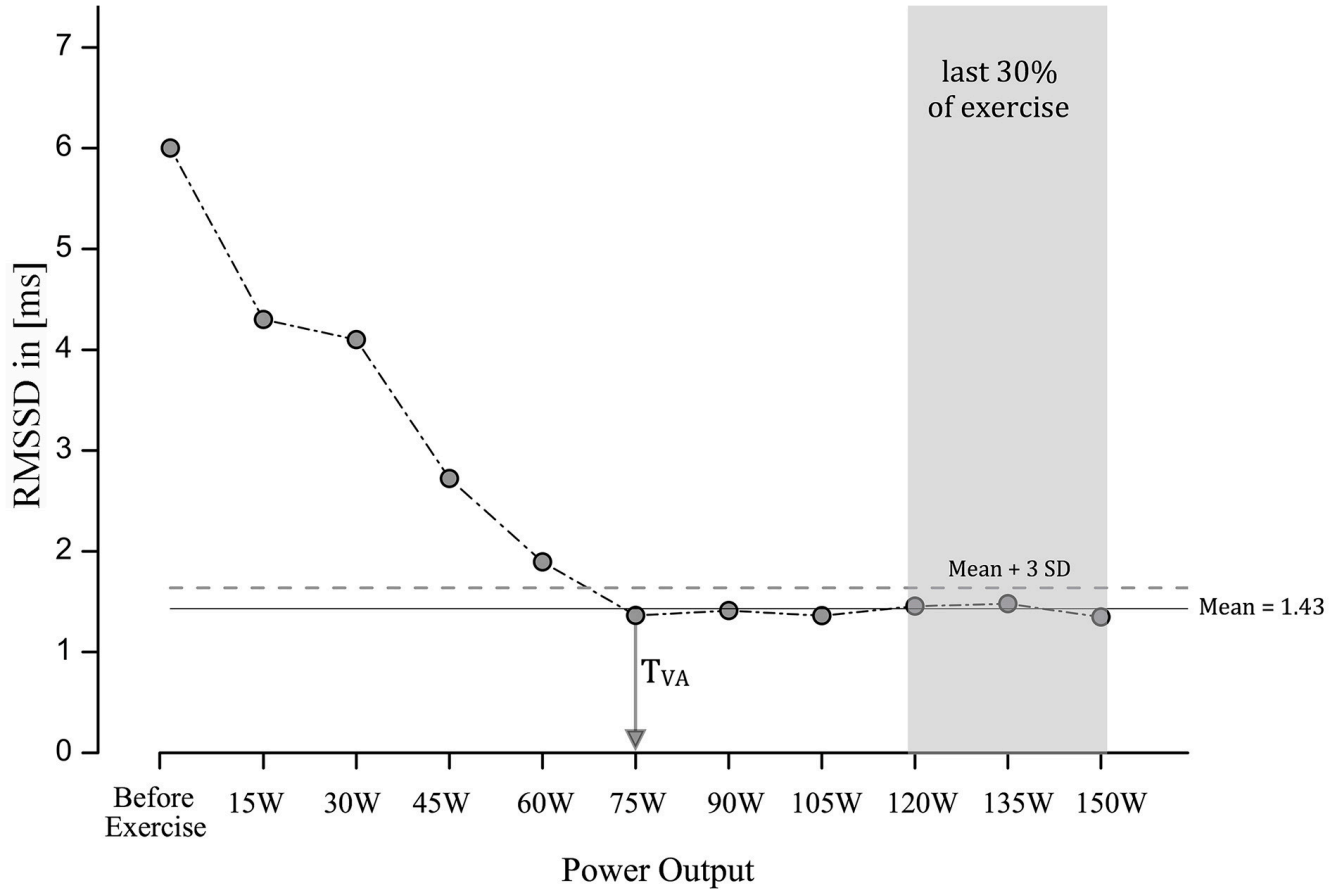
**The calculation of the vagal threshold is depicted using data from one subject for illustration**. The deflection point is the point in time at which no subsequent decline in heart rate variability occurs. DP was determined when the heart rate variability dropped below a threshold (mean value of the heart rate variability plus 3 times the standard deviation during the last 30% of the “exercise time”). In this time period, the decline in heart rate variability was completed and there were almost no variations in heart rate variability observed.

To characterize parasympathetic withdrawal, the vagal threshold (VT) was extracted separating an initial linear decline of RMSSD and a subsequent saturation phase. The deflection point (DP) is the point in time at which no subsequent decline in heart rate variability occurs (see Figure 2 for illustration), indicating the moment when parasympathetic activity has decreased and sympathetic activation will increase in such a way that almost no vagal modulation remains. DP was determined when the heart rate variability dropped below a threshold (mean value of the heart rate variability plus 3 times the standard deviation during the last 30% of the “exercise time”). In this time period during the last 30% of the test, the decline in heart rate variability was completed and there were almost no variations in heart rate variability for all subjects observed.

### MRI procedure

Data were collected on a 3 T whole body system equipped with a 12-element head matrix coil (MAGNETOM TIM Trio, Siemens). During the functional resting state MRI scan subjects were asked to keep their eyes closed during the whole session. Time series of 240 whole-brain ${\text{T}}_{2}^{*}$-weighted volume sets were acquired using a gradient-echo EPI sequence accelerated by parallel imaging using GRAPPA (TR = 2520 ms, TE = 30 ms, flip angle = 90°, inter-slice gap = 0.625 mm, GRAPPA factor = 2), each consisting of 45 contiguous transverse slices of 2.5 mm thickness covering the entire brain and lower brainstem. The matrix size was 88 × 84 pixels with an in-plane resolution of 2.5 × 2.5 mm^2^ corresponding to a field of view of 220 × 210 mm^2^. High-resolution anatomical T_1_-weighted volume scans (MP-RAGE) were obtained in sagittal orientation (TR = 2300 ms, TE = 3.03 ms, TI = 900 ms, flip angle = 9°, FOV = 256 mm, matrix = 256 × 256 mm, number of sagittal slices = 192, acceleration factor (PAT = 2) with isotropic resolution of 1 × 1 × 1 mm^3^.

### Physiological recordings during fMRI

The physiological recordings were assessed under similar conditions, i.e., lying in a MRI scanner before (T0) and after physical exercise intervention (T1). Finger pulse was acquired by a photoplethysmograph sensor attached to the distal phalanx of the left index finger. Respiratory activity was assessed by a strain gauge transducer incorporated in a belt tied around the chest approximately at the processus xiphoideus. Both signals were digitized at 500 Hz by the MR-compatible BIOPAC MP150 polygraph (BIOPAC Systems Inc., Goleta, CA, USA). The pulse signal was band-pass filtered between 0.5 and 5 Hz. Pulse waves were detected automatically and checked by visual inspection. This time series were post-processed by an adaptive filter algorithm described in detail by Wessel et al. (2000). Mean heart rate and root mean square of successive heart beat interval differences (RMSSD) were calculated according to the guidelines of the European Society of Cardiology (Malik et al., 1996).

### rs-fMRI preprocessing: Whole brain

The preprocessing of the whole brain (including brainstem and cerebellum) was performed using the SPM12 (http://www.fil.ion.ucl.ac.uk/spm), and AFNI (http://afni.nimh.nih.gov/afni/) software packages. The first five images were discarded to obtain steady-state tissue magnetization. Preprocessing included 3D motion correction, i.e., rigid body realignment to the mean of all images. It was ensured that head movement was below 3 mm and 3° for each participant. Subsequently, a slice timing correction was performed to ensure that the data on each slice corresponded to the same point in time. Afterwards, a within-subject registration was performed between functional and anatomical images. Using SPM12 the co-registered anatomical images were segmented and functional images were then spatially normalized to the MNI space using spatial normalization parameters estimated during the segmentation process. The whole-brain data were smoothed using a Gaussian filter of 6 mm FWHM. Further additional preprocessing steps were (i) removal of lineal and quadratic trends, (ii) temporal band-pass filtering, retaining frequencies in the 0.01–0.08 Hz band, (iii) removal by multiple regression of several sources of variance, i.e., head-motion parameter, global signal, CSF and white matter signal (Cordes et al., 2001; Murphy et al., 2009).

### rs-fMRI preprocessing: Brainstem/cerebellum

To improve the normalization of the brainstem, midbrain and cerebellum for the group level random effects (RFX) analysis, data were normalized to the spatially unbiased infra-tentorial template (SUIT, version 3.1; Diedrichsen, 2006). Using the SUIT toolbox we applied the following preprocessing steps: (i) segmentation of the whole-brain image as implemented in SPM12, (ii) cropping of the image, retaining only the cerebellum, brainstem and midbrain, (iii) normalization with the DARTEL engine (Ashburner, 2007) that uses gray and white matter segmentation maps produced during brainstem/cerebellum isolation to generate a flowfield using Large Deformation Diffeomorphic Metric Mapping (LDDMM, Beg et al., 2005), and (iv) reslicing the image to a voxel size of 2 × 2 × 2 mm^3^. The normalized images were smoothed with the Gaussian filter of 4 mm FWHM. Linear and quadratic trends were removed. The data were temporally filtered with a band-pass, retaining frequencies in the 0.01–0.08 Hz band. Furthermore, the head-motion parameter and the global signal (derived from the whole brain including brainstem and cerebellum) were removed using multiple regression.

### Definition of the seed regions nuclei

Based on our initial hypothesis, two ROIs were defined as a sphere of 5 mm radius, which represented the anterior part of the hippocampus (aHC) on the left (*x* = −28, *y* = −12, *z* = −20) and right side (*x* = 28, *y* = −12, *z* = −20). The coordinates were selected based on the boundaries of aHC-pHC as proposed by Poppenk et al. (2013). Moreover, they were in accordance with coordinates recently reported by Adnan et al. (2015), who derived the coordinates from the cluster-based parcellation of the hippocampus using diffusion-weighted imaging (DWI).

### Statistical analysis

Functional connectivity analyses were carried out by correlating the regional BOLD signal time courses, which were extracted from the left and right aHC-ROIs, against all other voxels in the whole brain as well as separately against all voxels in the brainstem/cerebellum. Statistical tests on regional functional connectivity data were performed after applying Fisher z-transformation to the correlation maps. A two-way ANOVA was set up with the first factor Group (exercise vs. control group) and the second factor Time (before vs. after exercise intervention) to test for significant Group × Time interaction with regard to resting state functional connectivity (RSFC) of the anterior hippocampi on the whole-brain level as well as only to voxels in the brainstem/cerebellum as preprocessed with the SUIT toolbox. All comparisons were thresholded at the uncorrected voxel-level significance of *p* < 0.001 and at the FWE corrected cluster-level significance of *p* < 0.05. The significance of the association between changes in the functional connectivity of aHC (z-scores from the cluster of significant Group x Time interaction) and significant exercise-based changes in the autonomic parameters (MANOVA, Group x Time interaction and *post-hoc t*-test) was tested using SPSS (IBM SPSS Statistics for Windows, Version 22.0).

## Results

### Changes in fitness and cardiorespiratory parameter

As reported previously (Wagner et al., 2015) and depicted in the Table 1, we detected a significant increase of the power output at the anaerobic threshold (IAnT) of 11.4% (*p* < 0.001), the maximum power output P_*max*_ of 11.2% (*p* < 0.001), and VO_2max_ adjusted for body weight of 4.7% (*p* < 0.001) in the exercise group (EG), which were all highly significant within the Group (exercise group vs. control group) × Time (before vs. after intervention) interaction of the ANOVA (P_*max*_: *p* < 0.001, IAnT: *p* = 0.002) except for VO_2max_ (*p* = 0.07).

### Autonomic function at baseline and after the intervention during the fMRI session

**Figure 3:**
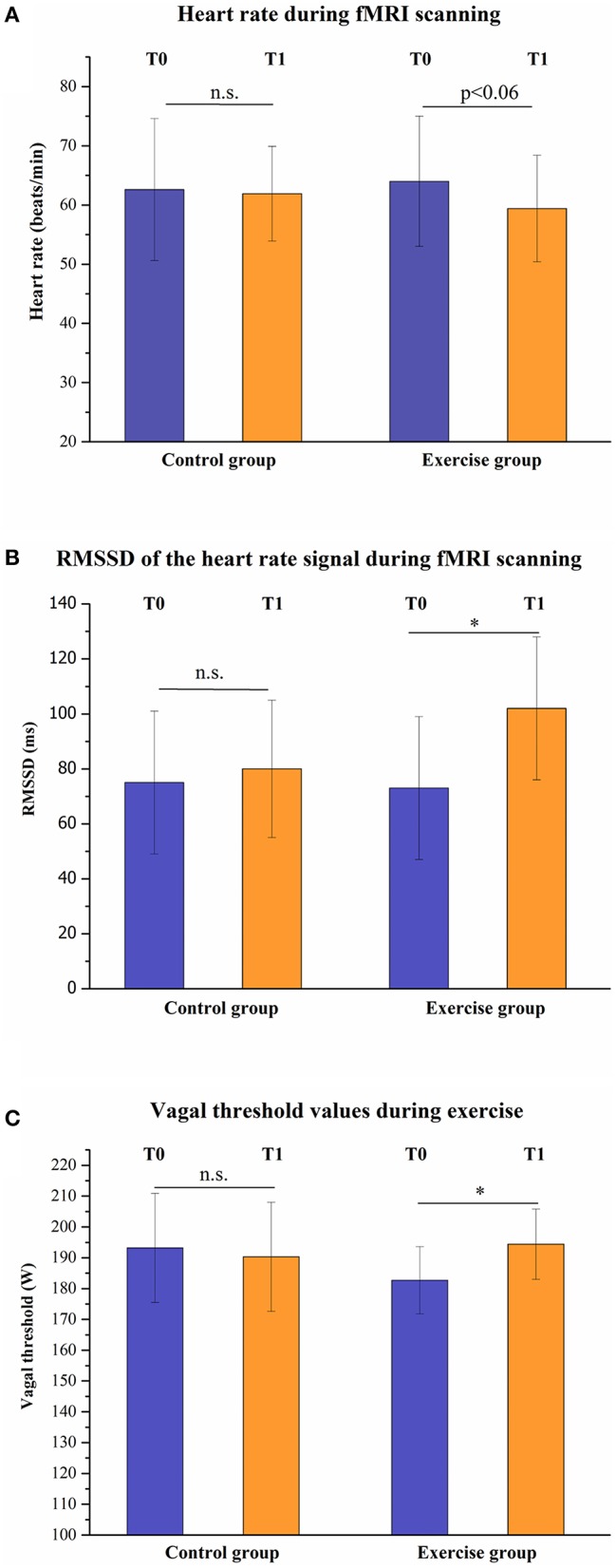
**Exercise-induced changes of heart rate (A), RMSSD (root mean square of successive heart beat interval differences, B) and vagal threshold (C) are displayed**. ^*^p < 0.05; n.s., not significant.

The overall MANOVA for the parameter heart rate during scanning, RMSSD during scanning and vagal threshold during exercise showed a trend toward significance for the Group (exercise group vs. control group) × Time (before vs. after intervention) interaction (*F* = 2.74; *p* < 0.06). As shown in Figure 3, no significant differences were observed in the *post-hoc t*-test for the control group between baseline (T0) and post-exercise (T1) for heart rate, parasympathetic time domain parameter RMSSD and vagal threshold. In contrast, comparing the baseline (T0) and the post-exercise (T1) of the exercise group, we observed a trend toward significance for a decrease in heart rate (*p* < 0.06) and a significant increase of vagal modulation as indicated by RMSSD (*p* < 0.026) and vagal threshold (*p* < 0.018).

### Resting state functional connectivity of the aHC: Whole brain

**Figure 4:**
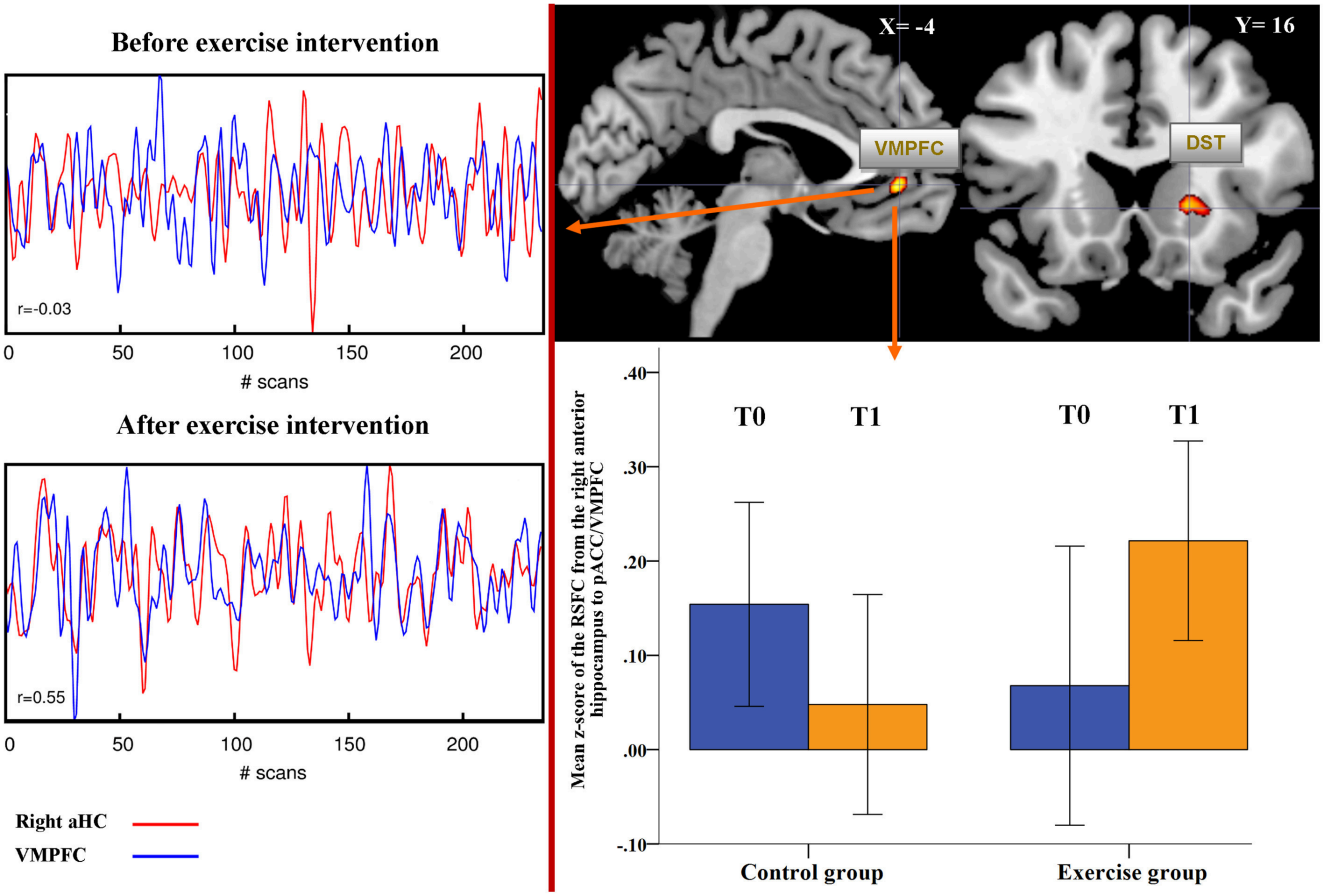
**Significant exercise-induced changes of the functional connectivity of the right aHC at the whole-brain level**. On the right-hand side: significant Group (exercise vs. control group) × Time (before vs. after intervention) interaction (*post-hoc t-test*) for the functional connectivity (FC) of the right anterior hippocampus (*p* < 0.001 uncorr, *p* < 0.05 cluster-level FWE corr.) at the whole-brain level. The box chart depicts significant changes in the FC between the right aHC and VMPFC/perigenual ACC (pACC). On the left-hand side: the correlations between the time series derived from the right aHC and the VMPFC/pACC are depicted in one subject before and after physical exercise intervention. DST, dorsal striatum; VMPFC, ventromedial prefrontal cortex; pACC, perigenual anterior cingulate cortex.

As illustrated in Figure 4, we observed a significant Group × Time interaction in the functional connectivity from the right aHC to a cluster comprising the ventromedial prefrontal cortex (VMPFC) and perigenual anterior cingulate cortex (pACC) (BA 10/32, *x* = −4, *y* = 44, *z* = −4, *t* = 4.72, *p* < 0.001, uncorr., cluster size = 64, *p* < 0.05, FWE corr.) and to the dorsal striatum, encompassing putamen and caudate (*x* = 18, *y* = 16, *z* = 2, *t* = 4.37, *p* < 0.001, uncorr., cluster size = 56, *p* < 0.05, FWE corr.). This result indicates an increase in the RSFC from the right aHC to these two regions in the exercise group (Figure 4). We further detected a significant Group × Time interaction with respect to the functional connectivity from the right aHC to precuneus (BA 31, *x* = 16, *y* = −64, *z* = 18, *t* = 4.36, *p* < 0.001, uncorr., cluster size = 160, *p* < 0.05, FWE corr.) and to cuneus (BA 18, *x* = 2, *y* = −90, *z* = 16, *t* = 4.13, *p* < 0.001, uncorr., cluster size = 66, *p* < 0.05, FWE corr.), indicating decreased RSFC from the right aHC to these two regions in the exercise group after intense training.

Testing for potential group difference in the RSFC of right aHC before the training intervention (T0), we did not detect significant differences except higher functional connectivity between the right aHC and precuneus (BA 31, *x* = 16, *y* = −66, *z* = 22, *t* = 4.39, *p* < 0.001, uncorr., cluster size = 89, *p* < 0.05, FWE corr.) in the exercise compared to the control group. After physical exercise (T1), a significantly stronger RSFC of the right aHC with VMPFC/pACC (*x* = −4, *y* = 44, *z* = −2, *t* = 4.56, *p* < 0.001, uncorr., cluster size = 51, *p* < 0.05, FWE corr.) and with the left ventrolateral prefrontal cortex (VLPFC; *x* = −36, *y* = 20, *z* = −22, *t* = 4.48, cluster size = 82, *p* < 0.05, FWE corr.) were observed in the exercise compared to the control group. There was no significant Group × Time interaction based on the RSFC from the left aHC at the whole-brain level.

### Resting state functional connectivity of the aHC: Brainstem/cerebellum

**Figure 5:**
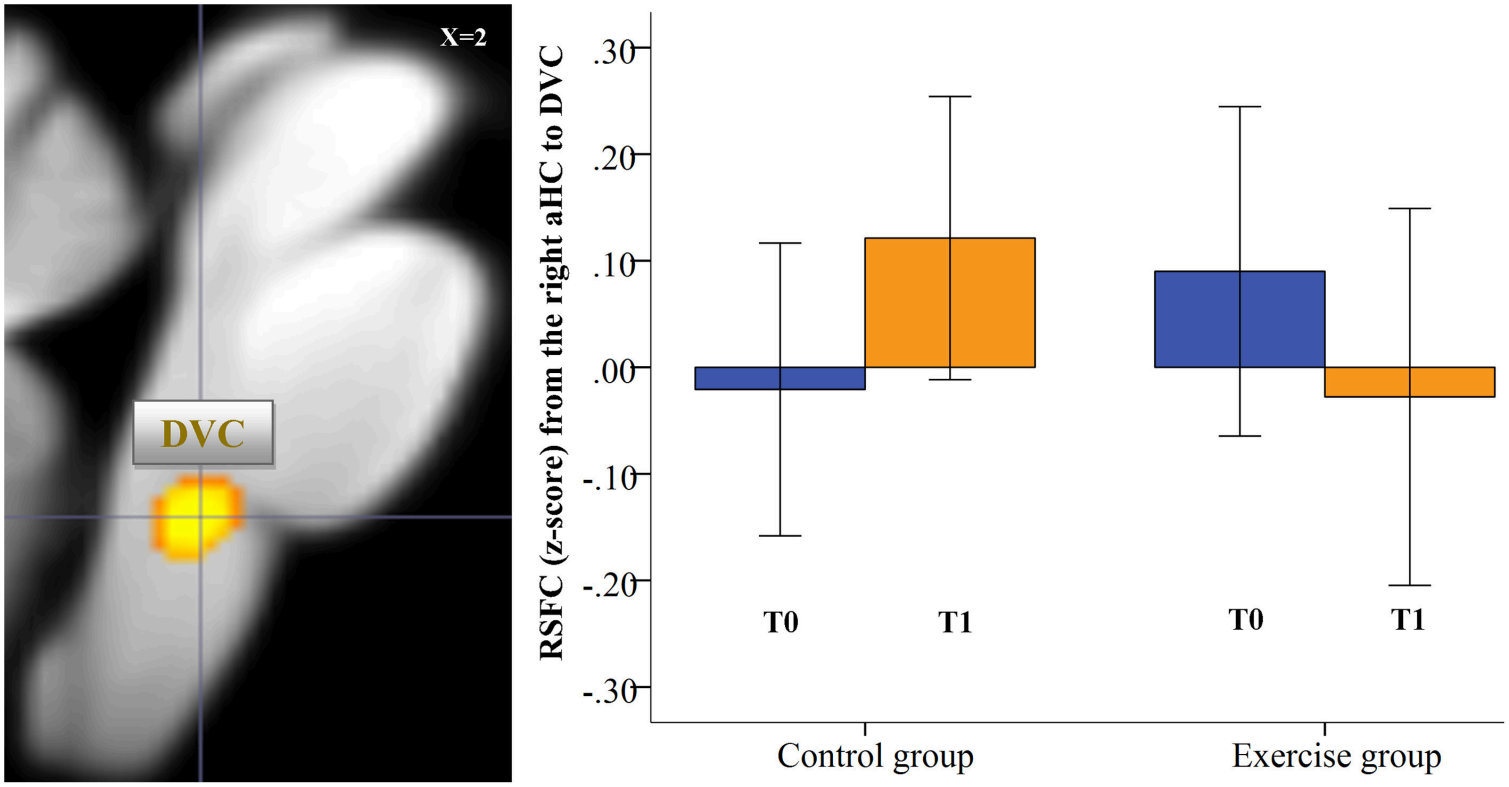
**Significant exercise-induced changes of the functional connectivity of the right aHC at the brainstem/cerebellum level**. Significant Group (exercise vs. control group) × Time (before vs. after intervention) interaction (*post-hoc t-test*) for the functional connectivity of the right anterior hippocampus (*p* < 0.001 uncorr, *p* < 0.05 cluster-level FWE corr.) at the brainstem/cerebellum level as preprocessed with the SUIT toolbox. The box chart depicts significant changes in the FC between the right aHC and a significant cluster located in the dorsomedial medulla, where a number of autonomic centers are located and which is in the vicinity of the previously described dorsal vagal complex (DVC, Blessing, 1997).

**Figure 6:**
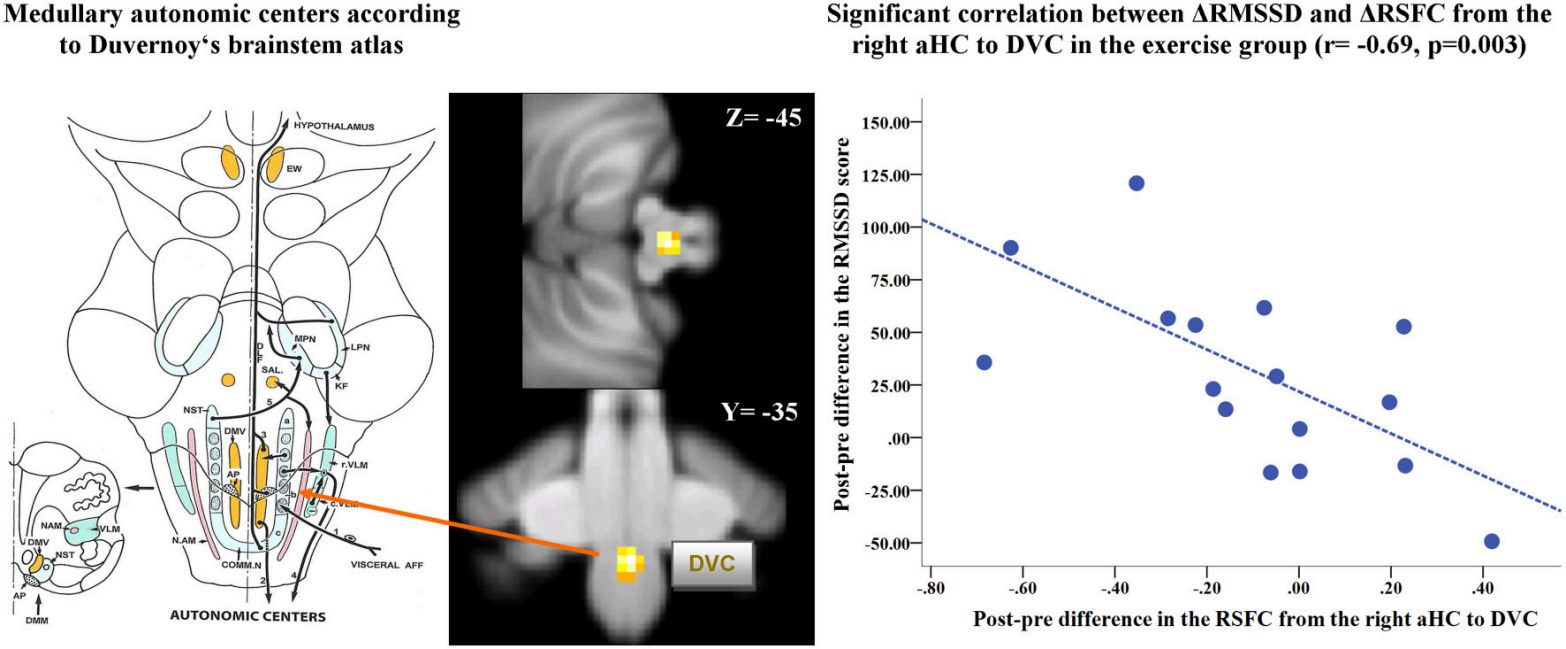
**Scatterplot of the significant negative correlation between exercise-induced changes in the functional connectivity from the right aHC to DVC and RMSSD**. On the right-hand side, the scatterplot illustrates the significant negative correlation in the exercise group between post-pre differences of functional connectivity of the right aHC to DVC and post-pre differences in RMSSD, which was measured during the resting state fMRI. RMSSD is considered as a parameter of heart rate variability, reflecting the integrity of vagus nerve-mediated autonomic control of the heart. On the left-hand side, a figure of the anatomical position of the autonomic medullary centers from the Duvernoy's Atlas of the Human Brain Stem and Cerebellum is depicted, corresponding to the significant cluster (DVC) of decreased function connectivity from the aHC after exercise. NST, nucleus of the solitary tract (a: rostral portion, b: midportion, c: caudal portion); COMM.N, commissural nucleus; DMV, dorsal motor nucleus of the vagus; AP, area postrema; DLF, dorsal longitudinal fasciculus; VLM, ventrolateral medullary center (r: rostral portion, c: caudal portion); N.AM, nucleus ambiguous; SAL, salivatory nucleus; MPN, medial parabrachial nucleus; LPN, lateral parabrachial nucleus; KF, nucleus of Kölliker-Fuse; EW, Edinger-Westphal. “With kind permission from Springer Science+Business Media: Duvernoy's Atlas of the Human Brain Stem and Cerebellum, Section III: Major Functions of the Human Brain Stem, 2009, page 115, Naidich, T.P., Duvernoy, H.M, Delman, B.N., Sorensen, A.G., Kollias, S.S., Haacke, E.M., Figure 3.13.”

Focusing on the brainstem/cerebellum only, a highly significant Group × Time interaction (Figure 5) resulted for the functional connectivity between the right aHC and a cluster located in the medial part of the medulla, which comprises a region (see Figure 6) including the nucleus of the solitary tract, nucleus ambiguous and dorsal nucleus of the vagus (*x* = 2, *y* = −36, *z* = −45, *t* = 6.35, *p* = 0.001, FWE corr., cluster size = 26, *p* < 0.05, FWE corr.) which is called the dorsal vagal complex (DVC) in animals (Blessing, 1997). We further detected a significant cluster located in the left cerebellum (*x* = −16, *y* = −36, *z* = −41, *t* = 5.39, *p* < 0.03, FWE corr., cluster size = 40, *p* < 0.05, FWE corr.). As depicted in Figure 5, the bar graphs of the functional connectivity between aHC and DVC indicate a decrease in the connectivity after exercise. We did not detect a significant Group × Time interaction in terms of increased RSFC between aHC and brainstem/cerebellum in the exercise group. Testing for potential group difference in the RSFC between right aHC and brainstem/cerebellum before the training intervention (T0), we did not detect significant differences. After the physical exercise intervention (T1), a significantly lower RSFC of the right aHC with DVC (*x* = 2, *y* = −36, *z* = −45, *t* = 4.95, *p* = 0.001, cluster size = 11) and with a cerebellar cluster (*x* = −16, *y* = −36, *z* = −41, *t* = 4.47, cluster size = 26, *p* < 0.05, FWE corr.) was observed in the exercise compared to the control group.

With respect to RSFC of the left aHC, we only observed a significant Group × Time interaction between left aHC and left cerebellum (*x* = −4, *y* = −42, *z* = −21, *t* = 4.54, *p* < 0.001, uncorr., cluster size = 58, *p* < 0.05, FWE corr.) as well as right cerebellum (*x* = 6, *y* = −44, *z* = −21, *t* = 4.36, *p* < 0.001, uncorr., cluster size = 30, *p* < 0.05, FWE corr.), indicating a decreased left aHC RSFC after exercise.

### Correlational analyses

In the exercise group, significant pre-post changes in heart rate and in the RMSSD, as assessed during the resting state fMRI, were correlated with the functional connectivity of the aHC to brain regions, which showed a significant Group × Time interaction. As shown in Figure 6, we only observed a highly significant negative correlation between increased RMSSD after exercise and decreased functional connectivity from the right aHC to DVC (*r* = −0.69, *p* = 0.003).

## Discussion

The anterior hippocampus has been associated amongst others with affective processing, such as stress, and anxiety regulation (Bannerman et al., 2004; Fanselow and Dong, 2010; Snyder et al., 2011). For example, lesions of the ventral (anterior) hippocampus have been shown to decrease anxiety-like behavior on a number of tasks (Fanselow and Dong, 2010). Long-term physical exercise and thus the degree of an individual's physical fitness influences not only bodily well-being and life-expectancy, but moreover the emotional state and cognitive functioning. It has been repeatedly shown that regular exercise possesses anxiolytic and antidepressant like effects both in rodents and humans (Duman et al., 2008; Herring et al., 2010; Gaudlitz et al., 2015; Zschucke et al., 2015). Some of these beneficial effects might be attributed to an increase in vagal modulation due to chronic exercise. The salutary effects of increased vagal function have been extensively described and are known to be very diverse. Thus, a range from cholinergic anti-inflammatory effects up to vagal modulations of emotional stimuli has been suggested (Ottani et al., 2009; Park and Thayer, 2014). However, the definite mechanisms underlying exercise induced increases of central vagal modulation are not well understood. Here, we hypothesized that the aHC which is subject to exercise-induced structural changes (Erickson et al., 2011; Wagner et al., 2015) might play a role in exercise induced adjustments of vagal function.

The high intensity protocol of the present study induced a significant shift of the autonomic balance toward vagal modulation after 8 weeks. We show that the parasympathetic time domain parameter RMSSD was increased during the second fMRI scanning session in the exercise group only. A further marker of vagal modulation (vagal threshold, VT) was chosen to indicate vagal function during exercise (Ostermann et al., 2013). To understand the concept of this parameter, it is important to comprehend that a reduction in efferent cardiac vagal activity allows an increase in cardiac output during low exercise intensity (Tulppo et al., 1998). Furthermore, progressive increases in exercise intensity lead to a critical point called the vagal threshold where further increases in exercise intensity cause negligible changes in vagal activity (Botek et al., 2010). According to Tulppo et al. (1998), vagal modulation disappears during exercise at the level of 50–60% VO_2max_. Assuming that vagal threshold is an indicator of vagal modulation closely related to physical fitness, we present evidence in the present study for an increase in vagal modulation during physical exercise. Thus, the exercise intervention led to a profound increase in vagal function in the exercising group.

To analyze putative central mechanisms underlying this increase in vagal modulation, we studied RSFC using fMRI technique. These analyses were carried out by correlating the regional time course, which was extracted from the right and left aHC, against all other voxels within the whole brain and especially within brainstem/cerebellum. In particular, we looked for differences of functional connectivity over time between the exercise and control group (Time x Group interactions). Interestingly, we observed with respect to cortical/subcortical regions an increase of functional connectivity from the right aHC to the VMPFC/perigenual ACC and to the dorsal striatum. We further detected decreased RSFC from the right aHC to precuneus and to cuneus. These very few cortical changes in RSFC are highly significant and specific.

It is well known that the VMPFC has extensive efferent projections to brain nuclei involved in cardio-vascular control in rodents (Verberne and Owens, 1998). It is therefore highly likely that the observed change in RSFC might closely be related to the increase in vagal modulation as observed in our study. Previous reports in animals describe that projections of the VMPFC synapse with the lateral hypothalamus and converge onto the nucleus tractus solitarii. Furthermore, an involvement with inhibitory synapses in the rostral ventrolateral medulla and excitatory synapses with the caudal ventrolateral medulla has been described (Resstel and Correa, 2006). Thus, activity in the VMPFC influences key areas of stress responses and cardiovascular regulation including baroreflex sensitivity (Guyenet, 2006). Interestingly, it has been reported that tasks that elicit a heart rate response (e.g., hand grip) were accompanied by reduced activation relative to baseline in the hippocampus and medial prefrontal cortex in humans (Shoemaker et al., 2012). Thus, observed changes of RSFC between hippocampus and medial prefrontal cortex are suggestive to serve as one mechanism responsible for increased vagal function after the exercise intervention. While we assume that altered RSFC to the dorsal striatum might be related to the motor component of regular training, interesting results have been reported with respect to the cuneus, which is the third cortical region showing altered RSFC to the right aHC after the exercise intervention. The cuneus is primarily involved in basic visual perception and emotional processing (Seiferth et al., 2009). Park et al. (2012) reported that participants with low resting heart rate variability showed heightened activity in the cuneus in response to fearful faces at high spatial frequency. The authors conclude that result might indicate that low heart rate variability (low vagal modulation) might be associated with hypervigilant physiological responses. We can only speculate that the observed decreased connectivity between aHC and cuneus might be associated with the degree of vagal modulation and influences physiological responses. However, this might be an interesting area for new research. This is of particular interest for exercise intervention studies, since patients with schizophrenia known to have low vagal modulation show greater activations in the middle occipital gyrus and the cuneus in response to neutral stimuli (Seiferth et al., 2008).

In addition to changes of RSFC of the aHC to cortical/subcortical regions, we observed a decrease in connectivity after exercise in an area in the brainstem encompassing the nucleus of the solitary tract, nucleus ambiguous and the dorsal nucleus of the vagus. According to the functional significance, we used the term introduced by Blessing (1997) DVC to describe this region. It is highly likely that the observed decrease in RSFC between aHC and DVC is closely related to increased vagal function since we observed a highly significant negative correlation between vagal modulation (increased RMSSD) and decreased functional connectivity from the right aHC to DVC. It is beyond the scope of this study to describe putative multisynaptic pathways involved in this interaction. However, previous studies found that ear lobe vagal stimulation leads to an increased activation in vagal nuclei and decreased hippocampal activity (Frangos et al., 2015). We therefore strongly suggest that the observed RSFC change between aHC and DVC might closely be related to the exercise-induced increase in vagal modulation. Moreover, it has been proposed that the DVC is a multifunctional reflex center of the autonomic nervous system which belongs to the few structures in the mammalian brain (such as the hippocampus and hypothalamus) showing adult neurogenesis (Bauer et al., 2005; Emsley et al., 2005; Kokoeva et al., 2005). Although the role of neurogenesis in the DVC is unknown, it might underlie long-term adaptations to exercise. Exercise associated changes in the hippocampus are thought to be mediated by the neurotrophic factor BDNF (brain derived neurotrophic factor) which is also involved in neuroplastic changes in the DVC region (Conner et al., 1997; Huang et al., 2014). Thus, future animal studies need to investigate whether this region is subject to exercise induced changes involved in increased vagal function.

Some limitations need to be addressed. First of all, our findings are confined to men only. In addition, our results were obtained at rest and we cannot transfer findings to vagal function during various tasks. Moreover, we focused in the present study on exercise-induced changes in the RSFC analysis of the anterior hippocampus based on existing studies and on our previous structural results. Since we did not investigate temporal dynamics (e.g., phase-delay) in terms of a causal relationship between aHC and functionally connected brain regions, the present findings need to be interpreted with caution. Regular physical exercise may also affect further brain areas (e.g., VMPFC) and thereby influencing functional connectivity.

Furthermore, we referred to the significant cluster in the lower brainstem as the DVC based on the comparison with the Duvernoy's Atlas of the Human Brain Stem and Cerebellum. However, as depicted in the Figure 6 on the left-hand side, a number of autonomic nuclei are situated in the medulla and our functional image resolution is not sufficient to differentiate between these small and closely spaced nuclei. Therefore, we anticipate future studies with increased spatial resolution will be able to confirm and to expand present results. In conclusion, we show evidence that exercise induced changes of RSFC of the aHC might be involved in increased vagal modulation. This evidence was found in the connectivity pattern of this region to neocortical and to brainstem regions. Further studies are needed to better understand the involvement of the aHC in autonomic regulation.

## Author contributions

KB: study coordinator, writing the manuscript, coordination of contributors. AS: Analysis of heart rate changes and study assistance in MRI department. MH: Training of participants and design of exercise intervention. FD: statistical analysis, preprocessing of resting state data. HG: Supervision of the exercise intervention, exercise intervention plan, writing of manuscript. GW: analysis of resting state data, study idea and enrollment, writing of the manuscript.

## Funding

Internal funds only.

### Conflict of interest statement

The authors declare that the research was conducted in the absence of any commercial or financial relationships that could be construed as a potential conflict of interest.

## References

[B1] Adnan A. Barnett A. Moayedi M. McCormick C. Cohn M. McAndrews M. P. (2015). Distinct hippocampal functional networks revealed by tractography-based parcellation. Brain Struct. Funct. 10.1007/s00429-015-1084-x. [Epub ahead of print]. 26206251

[B2] Ashburner J. (2007). A fast diffeomorphic image registration algorithm. Neuroimage 38, 95–113. 10.1016/j.neuroimage.2007.07.007 17761438

[B3] Bannerman D. M. Rawlins J. N. McHugh S. B. Deacon R. M. Yee B. K. Bast T.. (2004). Regional dissociations within the hippocampus–memory and anxiety. Neurosci. Biobehav. Rev. 28, 273–283. 10.1016/j.neubiorev.2004.03.004 15225971

[B4] Bauer S. Hay M. Amilhon B. Jean A. Moyse E. (2005). *In vivo* neurogenesis in the dorsal vagal complex of the adult rat brainstem. Neuroscience 130, 75–90. 10.1016/j.neuroscience.2004.08.047 15561426

[B5] Beg M. F. Miller M. I. Trouve A. Younes L. (2005). Computing large deformation metric mappings via geodesic flows of diffeomorphisms. Int. J. Comput. Vis. 61, 139–157. 10.1023/B:VISI.0000043755.93987.aa

[B6] Beissner F. Meissner K. Bär K. J. Napadow V. (2013). The autonomic brain: an activation likelihood estimation meta-analysis for central processing of autonomic function. J. Neurosci. 33, 10503–10511. 10.1523/JNEUROSCI.1103-13.2013 23785162 PMC3685840

[B7] Blessing E. M. Beissner F. Schumann A. Brunner F. Bär K. J. (2016). A data-driven approach to mapping cortical and subcortical intrinsic functional connectivity along the longitudinal hippocampal axis. Hum. Brain Mapp. 37, 462–476. 10.1002/hbm.23042 26538342 PMC6867561

[B8] Blessing W. W. (1997). The Lower Brainstem and Bodily Homeostasis. New York, NY: Oxford University Press.

[B9] Botek M. Stejskal P. Krejci J. Jakubec A. Gaba A. (2010). Vagal threshold determination. Effect of age and gender. Int. J. Sports Med. 31, 768–772. 10.1055/s-0030-1263141 20835977

[B10] Briggs G. G. Nebes R. D. (1975). Patterns of hand preference in a student population. Cortex 11, 230–238. 10.1016/S0010-9452(75)80005-0 1204363

[B11] Brum P. C. Da Silva G. J. Moreira E. D. Ida F. Negrao C. E. Krieger E. M. (2000). Exercise training increases baroreceptor gain sensitivity in normal and hypertensive rats. Hypertension 36, 1018–1022. 10.1161/01.HYP.36.6.1018 11116118

[B12] Castle M. Comoli E. Loewy A. D. (2005). Autonomic brainstem nuclei are linked to the hippocampus. Neuroscience 134, 657–669. 10.1016/j.neuroscience.2005.04.031 15975727

[B13] Cenquizca L. A. Swanson L. W. (2007). Spatial organization of direct hippocampal field CA1 axonal projections to the rest of the cerebral cortex. Brain Res. Rev. 56, 1–26. 10.1016/j.brainresrev.2007.05.002 17559940 PMC2171036

[B14] Clark K. B. Krahl S. E. Smith D. C. Jensen R. A. (1995). Post-training unilateral vagal stimulation enhances retention performance in the rat. Neurobiol. Learn. Mem. 63, 213–216. 10.1006/nlme.1995.1024 7670833

[B15] Clark K. B. Naritoku D. K. Smith D. C. Browning R. A. Jensen R. A. (1999). Enhanced recognition memory following vagus nerve stimulation in human subjects. Nat. Neurosci. 2, 94–98. 10.1038/4600 10195186

[B16] Conner J. M. Lauterborn J. C. Yan Q. Gall C. M. Varon S. (1997). Distribution of brain-derived neurotrophic factor (BDNF) protein and mRNA in the normal adult rat CNS: evidence for anterograde axonal transport. J. Neurosci. 17, 2295–2313. 9065491 10.1523/JNEUROSCI.17-07-02295.1997 PMC6573520

[B17] Cordes D. Haughton V. M. Arfanakis K. Carew J. D. Turski P. A. Moritz C. H.. (2001). Frequencies contributing to functional connectivity in the cerebral cortex in “resting-state” data. AJNR Am. J. Neuroradiol. 22, 1326–1333. 11498421 PMC7975218

[B18] Critchley H. D. (2005). Neural mechanisms of autonomic, affective, and cognitive integration. J. Comp. Neurol. 493, 154–166. 10.1002/cne.20749 16254997

[B19] Dampney R. A. (1994). Functional organization of central pathways regulating the cardiovascular system. Physiol. Rev. 74, 323–364. 8171117 10.1152/physrev.1994.74.2.323

[B20] Diedrichsen J. (2006). A spatially unbiased atlas template of the human cerebellum. Neuroimage 33, 127–138. 10.1016/j.neuroimage.2006.05.056 16904911

[B21] Dolcos F. Labar K. S. Cabeza R. (2004). Interaction between the amygdala and the medial temporal lobe memory system predicts better memory for emotional events. Neuron 42, 855–863. 10.1016/S0896-6273(04)00289-2 15182723

[B22] Duman C. H. Schlesinger L. Russell D. S. Duman R. S. (2008). Voluntary exercise produces antidepressant and anxiolytic behavioral effects in mice. Brain Res. 1199, 148–158. 10.1016/j.brainres.2007.12.047 18267317 PMC2330082

[B23] Emsley J. G. Mitchell B. D. Kempermann G. Macklis J. D. (2005). Adult neurogenesis and repair of the adult CNS with neural progenitors, precursors, and stem cells. Prog. Neurobiol. 75, 321–341. 10.1016/j.pneurobio.2005.04.002 15913880

[B24] Erickson K. I. Voss M. W. Prakash R. S. Basak C. Szabo A. Chaddock L.. (2011). Exercise training increases size of hippocampus and improves memory. Proc. Natl. Acad. Sci. U.S.A. 108, 3017–3022. 10.1073/pnas.1015950108 21282661 PMC3041121

[B25] Fanselow M. S. Dong H. W. (2010). Are the dorsal and ventral hippocampus functionally distinct structures? Neuron 65, 7–19. 10.1016/j.neuron.2009.11.031 20152109 PMC2822727

[B26] Frangos E. Ellrich J. Komisaruk B. R. (2015). Non-invasive access to the vagus nerve central projections via electrical stimulation of the external ear: fMRI evidence in humans. Brain Stimul. 8, 624–636. 10.1016/j.brs.2014.11.018 25573069 PMC4458242

[B27] Gaudlitz K. Plag J. Dimeo F. Strohle A. (2015). Aerobic exercise training facilitates the effectiveness of cognitive behavioral therapy in panic disorder. Depress. Anxiety 32, 221–228. 10.1002/da.22337 25515221

[B28] Guyenet P. G. (2006). The sympathetic control of blood pressure. Nat. Rev. Neurosci. 7, 335–346. 10.1038/nrn1902 16760914

[B29] Herring M. P. O'Connor P. J. Dishman R. K. (2010). The effect of exercise training on anxiety symptoms among patients: a systematic review. Arch. Intern. Med. 170, 321–331. 10.1001/archinternmed.2009.530 20177034

[B30] Huang T. Larsen K. T. Ried-Larsen M. Moller N. C. Andersen L. B. (2014). The effects of physical activity and exercise on brain-derived neurotrophic factor in healthy humans: a review. Scand. J. Med. Sci. Sports 24, 1–10. 10.1111/sms.12069 23600729

[B31] Kokoeva M. V. Yin H. Flier J. S. (2005). Neurogenesis in the hypothalamus of adult mice: potential role in energy balance. Science 310, 679–683. 10.1126/science.1115360 16254185

[B32] Lane R. D. McRae K. Reiman E. M. Chen K. Ahern G. L. Thayer J. F. (2009). Neural correlates of heart rate variability during emotion. Neuroimage 44, 213–222. 10.1016/j.neuroimage.2008.07.056 18778779

[B33] Ludowig E. Trautner P. Kurthen M. Schauer C. Bien C. G. Elger C. E.. (2008). Intracranially recorded memory-related potentials reveal higher posterior than anterior hippocampal involvement in verbal encoding and retrieval. J. Cogn. Neurosci. 20, 841–851. 10.1162/jocn.2008.20507 18201126

[B34] Malik M. Bigger J. Camm A. Kleiger R. (1996). Heart rate variability. Standards of measurement, physiological interpretation, and clinical use. Task Force of the European Society of Cardiology and the North American Society of Pacing and Electrophysiology. Eur. Heart J. 17, 354–381. 8737210

[B35] McAllen R. M. (1976). Proceedings: inhibition of the baroreceptor input to the medulla by stimulation of the hypothalamic defence area. J. Physiol. 257, 45P–46P. 948077

[B36] McAllen R. M. Spyer K. M. (1976). The location of cardiac vagal preganglionic motoneurones in the medulla of the cat. J. Physiol. 258, 187–204. 10.1113/jphysiol.1976.sp011414 940054 PMC1308967

[B37] Moser M. B. Moser E. I. Forrest E. Andersen P. Morris R. G. (1995). Spatial learning with a minislab in the dorsal hippocampus. Proc. Natl. Acad. Sci. U.S.A. 92, 9697–9701. 10.1073/pnas.92.21.9697 7568200 PMC40869

[B38] Murphy K. Birn R. M. Handwerker D. A. Jones T. B. Bandettini P. A. (2009). The impact of global signal regression on resting state correlations: are anti-correlated networks introduced? Neuroimage 44, 893–905. 10.1016/j.neuroimage.2008.09.036 18976716 PMC2750906

[B39] Murty V. P. Ritchey M. Adcock R. A. Labar K. S. (2010). fMRI studies of successful emotional memory encoding A quantitative meta-analysis. Neuropsychologia 48, 3459–3469. 10.1016/j.neuropsychologia.2010.07.030 20688087 PMC2949536

[B40] Nadel L. Hoscheidt S. Ryan L. R. (2013). Spatial cognition and the hippocampus: the anterior-posterior axis. J. Cogn. Neurosci. 25, 22–28. 10.1162/jocn_a_00313 23198887

[B41] Negrao C. E. Irigoyen M. C. Moreira E. D. Brum P. C. Freire P. M. Krieger E. M. (1993). Effect of exercise training on RSNA, baroreflex control, and blood pressure responsiveness. Am. J. Physiol. 265, R365–R370. 8368390 10.1152/ajpregu.1993.265.2.R365

[B42] Ostermann S. Herbsleb M. Schulz S. Donath L. Berger S. Eisentrager D.. (2013). Exercise reveals the interrelation of physical fitness, inflammatory response, psychopathology, and autonomic function in patients with schizophrenia. Schizophr. Bull. 39, 1139–1149. 10.1093/schbul/sbs085 22966149 PMC3756770

[B43] Ottani A. Giuliani D. Mioni C. Galantucci M. Minutoli L. Bitto A.. (2009). Vagus nerve mediates the protective effects of melanocortins against cerebral and systemic damage after ischemic stroke. J. Cereb. Blood Flow Metab. 29, 512–523. 10.1038/jcbfm.2008.140 19018269

[B44] Owens N. C. Verberne A. J. (2000). Medial prefrontal depressor response: involvement of the rostral and caudal ventrolateral medulla in the rat. J. Auton. Nerv. Syst. 78, 86–93. 10.1016/S0165-1838(99)00062-4 10789686

[B45] Park G. Moon E. Kim D. W. Lee S. H. (2012). Individual differences in cardiac vagal tone are associated with differential neural responses to facial expressions at different spatial frequencies: an ERP and sLORETA study. Cogn. Affect. Behav. Neurosci. 12, 777–793. 10.3758/s13415-012-0111-0 22815040

[B46] Park G. Thayer J. F. (2014). From the heart to the mind: cardiac vagal tone modulates top-down and bottom-up visual perception and attention to emotional stimuli. Front. Psychol. 5:278. 10.3389/fpsyg.2014.00278 24817853 PMC4013470

[B47] Pereira A. C. Huddleston D. E. Brickman A. M. Sosunov A. A. Hen R. McKhann G. M.. (2007). An *in vivo* correlate of exercise-induced neurogenesis in the adult dentate gyrus. Proc. Natl. Acad. Sci. U.S.A. 104, 5638–5643. 10.1073/pnas.0611721104 17374720 PMC1838482

[B48] Poppenk J. Evensmoen H. R. Moscovitch M. Nadel L. (2013). Long-axis specialization of the human hippocampus. Trends Cogn. Sci. 17, 230–240. 10.1016/j.tics.2013.03.005 23597720

[B49] Radna R. J. MaClean P. D. (1981). Vagal elicitation of respiratory-type and other unit responses in basal limbic structures of squirrel monkeys. Brain Res. 213, 45–61. 10.1016/0006-8993(81)91247-6 7237150

[B50] Resstel L. B. Correa F. M. (2006). Involvement of the medial prefrontal cortex in central cardiovascular modulation in the rat. Auton. Neurosci. 126–127, 130–138. 10.1016/j.autneu.2006.02.022 16603420

[B51] Ruit K. G. Neafsey E. J. (1990). Hippocampal input to a “visceral motor” corticobulbar pathway: an anatomical and electrophysiological study in the rat. Exp. Brain Res. 82, 606–616. 10.1007/BF00228802 1705519

[B52] Seiferth N. Y. Pauly K. Habel U. Kellermann T. Shah N. J. Ruhrmann S.. (2008). Increased neural response related to neutral faces in individuals at risk for psychosis. Neuroimage 40, 289–297. 10.1016/j.neuroimage.2007.11.020 18187342

[B53] Seiferth N. Y. Pauly K. Kellermann T. Shah N. J. Ott G. Herpertz-Dahlmann B.. (2009). Neuronal correlates of facial emotion discrimination in early onset schizophrenia. Neuropsychopharmacology 34, 477–487. 10.1038/npp.2008.93 18580874

[B54] Sheehan D. V. Lecrubier Y. Sheehan K. H. Amorim P. Janavs J. Weiller E.. (1998). The Mini-International Neuropsychiatric Interview (M.I.N.I.): the development and validation of a structured diagnostic psychiatric interview for DSM-IV and ICD-10. J. Clin. Psychiatry 59(Suppl.) 20, 22–33; quiz 34–57. 9881538

[B55] Shoemaker J. K. Norton K. N. Baker J. Luchyshyn T. (2015). Forebrain organization for autonomic cardiovascular control. Auton. Neurosci. 188, 5–9. 10.1016/j.autneu.2014.10.022 25458433

[B56] Shoemaker J. K. Wong S. W. Cechetto D. F. (2012). Cortical circuitry associated with reflex cardiovascular control in humans: does the cortical autonomic network “speak” or “listen” during cardiovascular arousal. Anat. Rec. (Hoboken). 295, 1375–1384. 10.1002/ar.22528 22848047

[B57] Snyder J. S. Soumier A. Brewer M. Pickel J. Cameron H. A. (2011). Adult hippocampal neurogenesis buffers stress responses and depressive behaviour. Nature 476, 458–461. 10.1038/nature10287 21814201 PMC3162077

[B58] Stegmann H. Kindermann W. Schnabel A. (1981). Lactate kinetics and individual anaerobic threshold. Int. J. Sports Med. 2, 160–165. 10.1055/s-2008-1034604 7333753

[B59] Strange B. A. Witter M. P. Lein E. S. Moser E. I. (2014). Functional organization of the hippocampal longitudinal axis. Nat. Rev. Neurosci. 15, 655–669. 10.1038/nrn3785 25234264

[B60] Sugawara J. Murakami H. Maeda S. Kuno S. Matsuda M. (2001). Change in post-exercise vagal reactivation with exercise training and detraining in young men. Eur. J. Appl. Physiol. 85, 259–263. 10.1007/s004210100443 11560079

[B61] Tannenholz L. Jimenez J. C. Kheirbek M. A. (2014). Local and regional heterogeneity underlying hippocampal modulation of cognition and mood. Front. Behav. Neurosci. 8:147. 10.3389/fnbeh.2014.00147 24834033 PMC4018538

[B62] Taylor E. W. Al-Ghamdi M. S. Ihmied I. H. Wang T. Abe A. S. (2001). The neuranatomical basis of central control of cardiorespiratory interactions in vertebrates. Exp. Physiol. 86, 771–776. 10.1111/j.1469-445X.2001.tb00043.x 11698972

[B63] Tulppo M. P. Makikallio T. H. Seppanen T. Laukkanen R. T. Huikuri H. V. (1998). Vagal modulation of heart rate during exercise: effects of age and physical fitness. Am. J. Physiol. 274, H424–H429. 9486244 10.1152/ajpheart.1998.274.2.H424

[B64] van Praag H. Kempermann G. Gage F. H. (1999). Running increases cell proliferation and neurogenesis in the adult mouse dentate gyrus. Nat. Neurosci. 2, 266–270. 10.1038/6368 10195220

[B65] Verberne A. J. Owens N. C. (1998). Cortical modulation of the cardiovascular system. Prog. Neurobiol. 54, 149–168. 10.1016/S0301-0082(97)00056-7 9481796

[B66] Viard A. Doeller C. F. Hartley T. Bird C. M. Burgess N. (2011). Anterior hippocampus and goal-directed spatial decision making. J. Neurosci. 31, 4613–4621. 10.1523/JNEUROSCI.4640-10.2011 21430161 PMC6622909

[B67] Wagner G. Herbsleb M. Cruz Fde L. Schumann A. Brunner F. Schachtzabel C.. (2015). Hippocampal structure, metabolism, and inflammatory response after a 6-week intense aerobic exercise in healthy young adults: a controlled trial. J. Cereb. Blood Flow Metab. 35, 1570–1578. 10.1038/jcbfm.2015.125 26082010 PMC4640322

[B68] Wessel N. Voss A. Mahlberg H. Ziehmann C. Voss H. U. Schirdewan A. (2000). Nonlinear analysis of complex phenomena in cardiological data. Herzschr Elektrophys 11, 159–173. 10.1007/s003990070035

[B69] Westerhaus M. J. Loewy A. D. (2001). Central representation of the sympathetic nervous system in the cerebral cortex. Brain Res. 903, 117–127. 10.1016/S0006-8993(01)02453-2 11382395

[B70] Ziegler G. Dahnke R. Yeragani V. K. Bär K. J. (2009). The relation of ventromedial prefrontal cortex activity and heart rate fluctuations at rest. Eur. J. Neurosci. 30, 2205–2210. 10.1111/j.1460-9568.2009.07008.x 20128855

[B71] Zschucke E. Renneberg B. Dimeo F. Wustenberg T. Strohle A. (2015). The stress-buffering effect of acute exercise: evidence for HPA axis negative feedback. Psychoneuroendocrinology 51, 414–425. 10.1016/j.psyneuen.2014.10.019 25462913

